# Preparation of MgF_2_ Coatings on AZ31 Mg Alloy in Micro-Arc Oxidation Process Based on the Solubility Product Rule

**DOI:** 10.3390/ma18122717

**Published:** 2025-06-09

**Authors:** Hao Wang, Yifeng Yang, Cancan Liu, Xuchen Lu

**Affiliations:** College of Materials Science and Engineering, Nanjing Tech University, Nanjing 211800, China; 202261203312@nitech.edu.cn (H.W.); 202261103119@njtech.edu.cn (Y.Y.); 202361203237@njtech.edu.cn (X.L.)

**Keywords:** micro-arc oxidation, KF, NH_4_F, solubility product rule, phase composition

## Abstract

This work mainly explores whether the solubility product principle has a guiding role in regulating the composition of micro-arc oxidation (MAO) coatings. The MAO process was conducted on AZ31 Mg alloy in silicate electrolyte. Varying amounts of Potassium fluoride (KF) and Ammonium fluoride (NH_4_F) were separately added to the basic electrolyte to regulate the OH^−^ and F^−^ contents in the electrolyte. The microstructure, phase composition and corrosion resistance of the MAO coatings prepared in different electrolytes were analyzed. Results showed that regardless of KF content, MgO was the main component for the MAO coatings obtained in electrolytes with KF. This was because the addition of KF not only elevated the F^−^ concentration in the electrolyte but also enhanced the OH^−^ concentration as a result of F^−^ hydrolysis. Based on the solubility product constants (Ksp) of MgO and MgF_2_, a relatively lower concentration of Mg^2+^ was sufficient for the formation of MgO. Hence, Mg^2+^ consistently exhibited preferential reactivity with OH^−^, leading to the formation of MgO. The findings of the study demonstrated that the presence of KF electrolyte resulted in an enhancement of conductivity and an increase in the concentration of OH^−^. Conversely, the growth rate of the coating was observed to be low, and the coating-forming phases of the coating were identified as MgO and Mg_2_SiO_4_, and the coating had better corrosion resistance. NH_4_F electrolyte with the increase in NH_4_F concentration, conductivity decreases and then increases, OH^−^ concentration decreases, the growth rate of the coating is faster, the concentration of F^−^/OH^−^ is higher, the coating-forming phase is transformed into MgF_2_, and the corrosion resistance of the coating is reduced.

## 1. Introduction

Magnesium (Mg) alloys have the advantages of high specific strength and low density, but the standard electrode potential of Mg is extremely negative, resulting in extremely poor corrosion resistance of Mg alloys, which severely limits the application of Mg alloys [1,2]. Consequently, surface treatment techniques are employed to enhance the corrosion resistance of Mg alloys. Micro-arc oxidation (MAO) technology has become a prevalent method for the surface treatment of Mg alloys, owing to its simplicity and environmental sustainability. This process entails the in situ growth of a ceramic coating on the surface of the base metal through a range of reactions, including electrochemical, thermochemical, plasma chemical, and others [3,4,5]. Research indicates that the composition of the MAO coating is influenced by various factors, particularly the electrolyte composition [6,7].

Alkaline electrolytes are divided into three types depending on the main salt, including phosphate, silicate and aluminum electrolytes [8,9,10]. In alkaline aqueous solution, it is unavoidable that reactive oxygen species are involved in the reaction at the anode to generate MAO coating. Therefore, the main components of Mg alloy MAO coatings in aqueous systems contain the base metal oxide (MgO). However, MgO is susceptible to hydrolysis in corrosive media, resulting in suboptimal long-term corrosion performance of MAO coatings on Mg alloys [11,12]. In order to increase the content of stabilizing compounds in MAO coatings, many scholars have attempted to improve the electrolyte system by adding special additives (Na_2_WO_4_, KF, K_2_ZrF_6_, K_2_TiF_6_, etc.) [13,14,15,16] or ceramic particles (HAP, Al_2_O_3_, ZrO_2_, TiO_2_, etc.) [17,18,19,20]. At the same time, some of these additives are not effective, and the electrolyte components are complex, and the electrolyte system is unstable.

For Mg substrates, fluoride coatings (MgF_2_) have better chemical stability and biocompatibility than oxide coatings (MgO) [21,22]. Wang et al. [23] showed that the introduction of NaF led to the formation of fluorinated nanolayers at the interface of the substrate and the coating, and that the increase in the concentration of NaF in the electrolyte increased the thickness of the coating and the content of MgF_2_. Liu et al. [24] reported that KF can reduce anodic dissolution on the surface of the Mg matrix by surface passivation. This process involves a competitive relationship between Mg^2+^ and OH^−^ and F^−^ to form insoluble compounds. The study by Chen et al. [25] hypothesized the potential reaction of fluoride ions in the MAO process and explained the mechanism of fluoride ion involvement in coating growth.

MAO coatings with adequate corrosion resistance can be prepared on the surface of magnesium alloys by the addition of various fluoride additives. However, fluoride coatings (MgF_2_) are less prevalent, and oxide coatings (MgO) predominate [26,27,28,29]. In this paper the formation of fluoride coatings on magnesium alloys under alkaline conditions has been further investigated. Based on the solubility product rule, different fluorine additives (KF, NH_4_F) are added to the electrolyte composed of sodium silicate and sodium hydroxide to change the concentrations of F^−^ and OH^−^ in the electrolyte, and a coating mainly composed of MgF_2_ is prepared. The present study investigates the effects of fluoride and hydroxide ions on the tissue characteristics, growth behavior, and corrosion resistance of the coatings.

## 2. Materials and Methods

### 2.1. Coating Preparation

**Figure 1:**
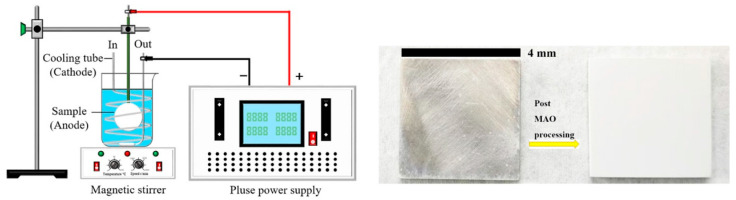
Schematic Diagram of MAO Device and Comparison of AZ31 Magnesium Alloy Before and After MAO Treatment.

AZ31 magnesium alloy specimens with dimensions of 40 mm × 40 mm × 4 mm were selected for the experimental study. (nominal composition in wt.%: Al 2.60, Zn 0.89, Mn 0.28 and balance Mg). The surface of each sample was polished with SiC papers up to 2000 grit and cleaned ultrasonically in ethanol. Figure 1 shows a schematic diagram of the experimental setup and sample images before and after the experiment.

MAO treatment (JCL-AMOZ10, CDJCL, China) was conducted utilizing a pulsed unipolar power supply (10 kW) in the constant current mode. The temperature of the electrolyte was maintained at 20 ± 4 °C by a cooling and stirring system. The electrical parameters of the MAO process were set as follows: current density 2 A/dm^2^, frequency 500 Hz, duty cycle 20%, and processing time 15 min. The base electrolyte was composed of 15 g/L Na_2_SiO_3_·9H_2_O and 5 g/L NaOH. Subsequently, KF·2H_2_O and NH_4_F were separately added to the base electrolyte at varying concentrations, namely 0.042 mol/L, 0.071 mol/L, 0.1 mol/L, 0.129 mol/L, 0.158 mol/L, 0.187 mol/L, and 0.216 mol/L. The electrolyte should be stirred with a magnetic stirrer for a period of time exceeding 30 min, the properties of the electrolyte were measured using a pH meter (INASE Scientific Instrument, PHS-25, China) and a conductivity meter (MEACON, DDS-11A, China) at room temperature (25 ± 2 °C).

The samples were expressed as 0.042 mol/L, 0.071 mol/L, 0.1 mol/L, 0.129 mol/L, 0.158 mol/L, 0.187 mol/L, and 0.216 mol/L (KF/NH_4_F), in that order, according to the content of KF and NH_4_F in the electrolytes.

### 2.2. Coating Characterization

The micro-morphology of the coatings was observed by a scanning electron microscope (SEM, JEOL, JSM-7900F, Japan) equipped with an energy dispersive X-ray spectrometer (EDS, JEOL, JSM-IT500A, Japan), and the surface structure analysis of the coating was conducted using the secondary electron imaging mode, while the cross-sectional microstructure analysis employed the backscattered electron mode. The coating phase was determined by X-ray diffraction (XRD, D/Max-2400, Japan), and the measurement was performed in grazing incidence mode with a grazing angle set at 2°, a scanning range of 2θ from 10° to 90°, a step size of 0.02°, and a scanning speed of 10°/min. The thickness of the coating was measured using an eddy current thickness gauge (FMP20, Fischer AG, Germany). The microporous structure of the coating was quantitatively characterized using ImageJ 1.8 software. The emission spectra of the discharge sparks during the MAO process were studied using an optical emission spectrometer (OES, Ideaooptics PG2000 Pro, China), background light interference was eliminated during testing, with the probe positioned parallel to and 3 cm away from the sample surface. The electrochemical corrosion behavior of the sample was evaluated using an electrochemical workstation (Auto-lab PGSTAT302 N, Switzerland) through a conventional three-electrode cell system. The saturated calomel electrode served as a reference electrode with the platinum electrode as a counter electrode. The potentiodynamic polarization curves were tested at a sweep rate of 10 mV/s after 1 h of immersion.

## 3. Results

### 3.1. Discharge Behavior of MAO in Electrolytes with Different Fluorine Additives

**Figure 2:**
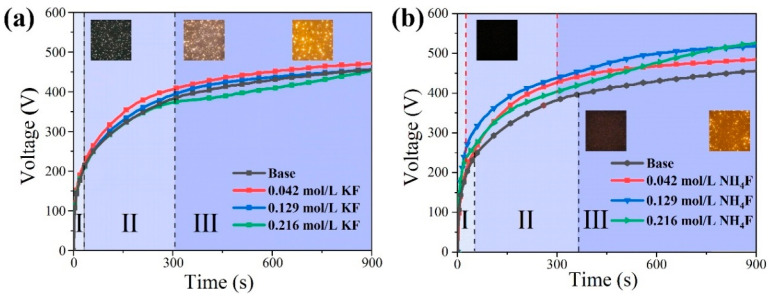
Voltage-time curves during MAO in electrolytes with different fluorine additives: (**a**) KF (**b**) NH_4_F.

As illustrated in Figure 2, the operating voltage versus time curves for the coating preparation process in different electrolytes are demonstrated. Table 1 presents the conductivity and pH values of the various electrolytes. From Table 1, it can be seen that in KF electrolyte the pH and conductivity increase with the increase in KF concentration, and in NH_4_F electrolyte the pH decreases continuously with the increase in NH_4_F concentration, and the conductivity decreases first and then increases. This phenomenon is primarily due to the fact that KF is a strong electrolyte, fully ionized in aqueous solution. Conversely, F^−^ is a weak acidic ion that undergoes hydrolysis, resulting in a slight increase in the pH of the electrolyte. The ionization of NH_4_F occurs during its dissolution in water; however, the degree of ionization is incomplete. Consequently, the hydrolysis of F^−^, along with the consumption of OH^−^ by the generation of NH_3_·H_2_O with OH^−^ in an alkaline electrolyte, results in a reduction in pH and an increase in conductivity. As demonstrated in Figure 2, stages I, II, and III correspond to the three phases of MAO (anodic oxidation, spark discharge, and micro-arc discharge), and it is evident that conductivity and breakdown voltage exhibit an inverse relationship, indicating that an increase in conductivity results in a decrease in the voltage required for breakdown to occur.

**Figure 3:**
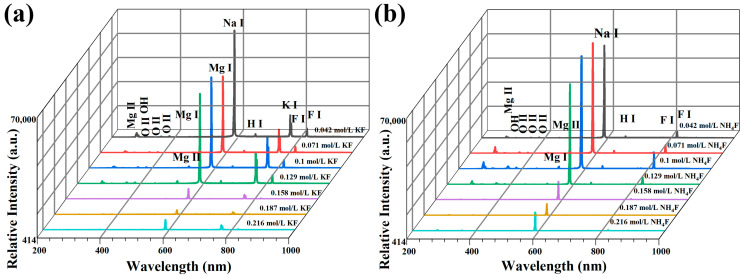
OES spectra of MAO in electrolytes with different fluorine additives at 15 min: (**a**) KF (**b**) NH_4_F.

Figure 3 illustrate the OES patterns in various electrolytes, respectively. As demonstrated in Figure 2, the voltage at each stage of MAO in the KF electrolyte is lower compared with that in the NH_4_F electrolyte. However, the spark is relatively larger, and the yellow spark at the stage of MAO is larger, but the position of the spark is relatively fixed. The underlying reason for this phenomenon is attributable to the elevated OH^−^ concentration present within the KF electrolyte. This elevated concentration gives rise to a substantial increase in conductivity, leading to a sharp rise in energy levels within the discharge channel. Consequently, the coating-forming reaction experiences a notable intensification, resulting in an augmentation of reactions occurring on the anode surface. The OES spectrum in Figure 3 exhibits characteristic spectral lines of Mg, O, Na, K, H, and F atoms from both the electrolyte and substrate during anodic luminescence, where labels I and II represent neutral atoms and singly ionized ions, respectively. This indicates that both the metal substrate and electrolyte components participate in the micro-arc discharge process during magnesium alloy MAO [30]. After the F^−^ concentration of 0.129 mol/L, the OES spectral line intensities were greatly weakened. Combined with Figure 2, it can be seen that with the increase in KF concentration, large sparks appeared on the anode surface in the late stage of MAO, and the spectral line intensities were related to the density of the discharged sparks as well as the intensity of illumination, and the fiber-optic spectrometer probe was very small (14 × 14 μm^2^), so that a small number of large sparks were not necessarily detectable by the fiber-optic probe, and the spectral line intensities were weakened accordingly. The intensity of the spectral lines decreases accordingly. Conversely, an increase in NH_4_F concentration results in the presence of only sporadic, diminutive sparks during the late stage of MAO. These sparks exhibit reduced illuminance and a diminished intensity of OES characteristic spectral lines.

### 3.2. Microstructure of the Coating in Electrolyte with Different Fluorine Additives

**Figure 4:**
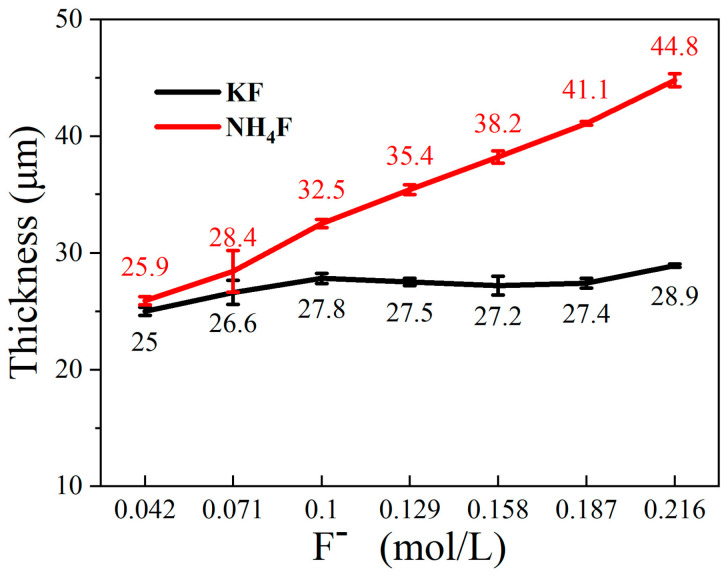
Thickness of MAO coating in electrolytes with different fluorine additives.

Figure 4 shows the thickness of the MAO coating in electrolyte with different fluorine additives. In the KF electrolyte, the KF concentration was increased from 0.042 mol/L to 0.216 mol/L. Concurrently, the coating thickness grew from 25 μm to 28.9 μm. However, the coating growth was inhibited. This phenomenon can be attributed to the elevated electrolyte conductivity resulting from increasing KF concentrations, the substantial pH of the electrolyte, the intensified MAO reaction, the accelerated dissolution rate of the coating, and the retarded growth rate of the coating. The coating thickness exhibited an increase from 25.9 μm to 44.8 μm in the NH_4_F electrolyte. This is because with the decrease in NH_4_F electrolyte conductivity, pH, the increase in electrolyte resistance, and the higher operating voltage in the constant current mode, the anions in the electrolyte move to the anode and form a coating under the action of stronger electric field, and the coating growth rate is not affected.

**Figure 5:**
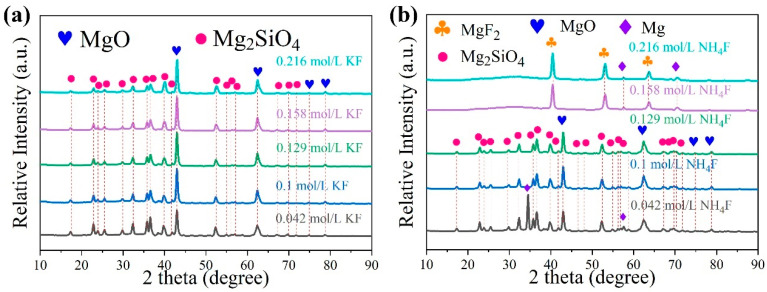
XRD patterns of MAO coatings in electrolytes with different fluorine additives: (**a**) KF (**b**) NH_4_F.

Figure 5 shows the XRD patterns of the MAO coating prepared in electrolyte with different fluorine additives. The results demonstrated that the alteration in the KF concentration within the KF electrolyte exhibited no impact on the composition of the components present within the coating. The MAO coating was predominantly composed of MgO and Mg_2_SiO_4_, and no peak corresponding to MgF_2_ was detected. Combined with the surface element contents of MAO coatings in the KF electrolyte in Table 2, it can be seen that the F concentration increases from 0.94 wt.% to 5.43 wt.% as the KF concentration increases, and the amount of F in the coatings is consistently lower. The formation of minor quantities of MgF_2_ may be associated with the solubility product (Ksp) of Mg(OH)_2_ and MgF_2_, which are 5.16 × 10^−11^ and 5.61 × 10^−12^ [31], respectively. In the process of MAO, the anions in the electrolyte (SiO_3_^2−^, OH^−^, F^−^) enter the discharge channel under the action of a strong electric field, and a series of complex electrochemical, thermochemical, and plasma chemical reactions take place after mixing with the molten metal in the channel. Meanwhile, due to the high concentration of OH^−^ in the electrolyte and the smaller Ksp of Mg(OH)_2_, SiO_3_^2−^ has a strong selective adsorption; thus, the main coating-forming phases are MgO and Mg_2_SiO_4_. In the NH_4_F electrolyte, an increase in NH_4_F concentration was observed to result in a shift in the predominant coating-forming substances of the coating from MgO and Mg_2_SiO_4_ to MgF_2_. The spectra of MgF_2_, as depicted in Figure 4, manifested as a package peak at 27.3°, while the spectral peaks of MgO and Mg_2_SiO_4_ disappeared, indicating a decline in conductivity within the electrolyte, This decline was accompanied by a reduction in the intensity of the spark and a decrease in the crystallinity of the coatings. In combination with the surface element content of MAO coating in NH_4_F electrolyte as presented in Table 3, an increase in F concentration from 1.39 wt.% to 20.91 wt.% is observed, whilst O and Si decrease from 35.25 wt.% and 18.91 wt.% to 23.51 wt.% and 14.53 wt.%, respectively. This indicates that the F−content in the electrolyte is continuously increasing, the OH^−^ concentration is continuously decreasing, and the MgF_2_ thermodynamic stability is strong, and the coating composition shift is dominated by MgF_2_.

**Figure 6:**
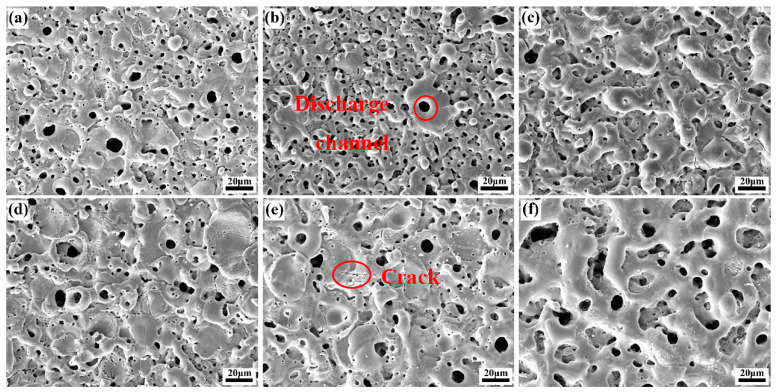
Surface morphology of MAO coating in electrolytes with different fluorine additives: (**a**–**c**): 0.042, 0.129, 0.216 mol/L KF; (**d**–**f**): 0.042, 0.129, 0.216 mol/L NH_4_F.

The microscopic morphology of the coating surface prepared in electrolyte with different fluorine additives is shown in Figure 6. When considered in conjunction with Table 4, which details the mean surface pore size and porosity in electrolyte with different fluorine additives, it becomes evident that the coating surfaces manifest the archetypal microporous structure of MAO coating. As asserted by Hussein et al. [32], three different types of discharges occur during MAO discharge, including oxide–electrolyte interface discharges at the top of the coating (type A), metal matrix–oxide interface discharges (type B), and deep hole and crack discharges in the coating (type C). The uniformity of the coating surface in the KF electrolyte is good, there are obvious pores on the surface of the coating, the average size of the micropores is small, and the MAO process is dominated by B-type discharge. In the NH_4_F electrolyte, at a lower NH_4_F concentration, the coating thickness is thinner and B-type discharge is the main form of discharge, while A- and C-type discharge are suppressed, and at higher NH_4_F concentration, the coating thickness is increased and the probability of A- and C-type discharge is higher than that of B-type discharge, which is manifested by the presence of fine and shallow micropores and bulging large oxides on the surface of the coating, in spite of the higher intensity of B-type discharge.

**Figure 7:**
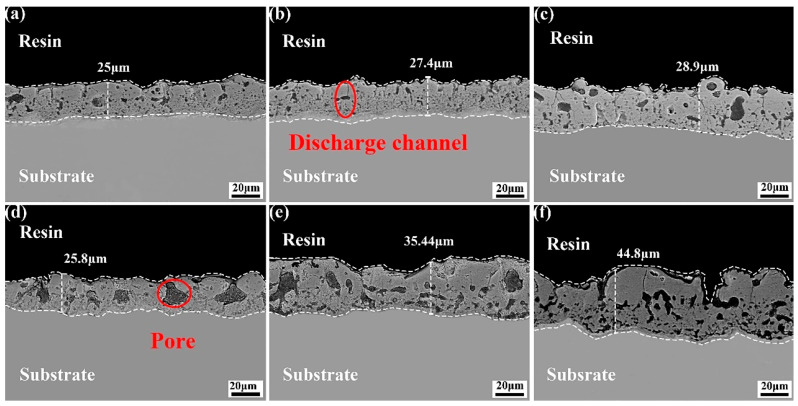
Cross-sectional morphology of MAO coating in electrolytes with different fluorine additives: (**a**–**c**): 0.042, 0.129, 0.216 mol/L KF; (**d**–**f**): 0.042, 0.129, 0.216 mol/L NH_4_F.

The cross-sectional morphology of the coatings prepared in electrolyte with different fluorine additives is illustrated in Figure 7. In the KF electrolyte, with the increase in KF concentration, the spark discharge on the surface of the specimen is more intense, and more energy is applied to the surface of the specimen, so that the degree of coating-forming reaction in the discharge channel increases, and more bubbles generated by the reaction cannot be overflowed in time to be wrapped in the coating, and the supply of coating-forming material is insufficient, resulting in the reduction in the amount of molten material generated by the MAO reaction deposited in the coating, and there are larger-sized pores in the coating, and the coating thickness is not significantly increased. Holes appear in the coating, and the thickness of the coating has not been greatly improved. With regard to the NH_4_F electrolyte, with the increase in NH_4_F concentration, the spark discharge on the specimen surface is weakened, and the main type of discharge changes from the B type to A type and C type, with more microporous defects in the inner layer and the surface sparse layer; the coating growth is dominated by deposition, the surface uniformity decreases, and there are micropores formed by C type discharges.

### 3.3. Effect of Different Fluoride Additives on the Corrosion Resistance of Coatings

**Figure 8:**
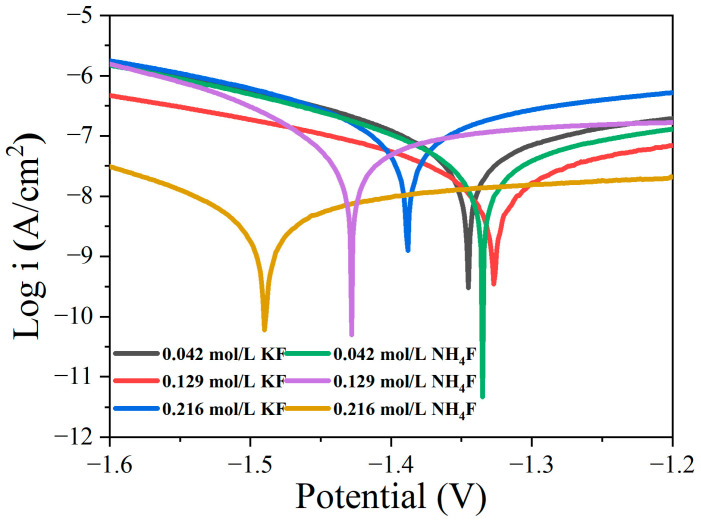
Potentiodynamic polarization curves of MAO coatings in electrolytes with different fluorine additives after being immersed in a 3.5 wt.% NaCl solution for 1 h.

Figure 8 shows the potentiodynamic polarization curves of coatings in electrolytes with different fluorine additives, and the fitting results are shown in Table 5. The higher the corrosion potential is, the more stable the coating is. The corrosion current density reflects the corrosion rate of the coating. The smaller the corrosion current density, the more corrosion resistant the coating is. The corrosion potential (E_corr_) and corrosion current density (i_corr_) of the coating in 0.216 mol/L NH_4_F electrolyte were −1486 mV and 2.45 × 10^−9^ A/cm^2^, respectively. Compared with the corrosion potential and corrosion current density of other coatings, the corrosion potential is relatively negative, and the corrosion current density is small. The corrosion potential is generally related to the thermodynamic stability of the material, which may be related to the decrease in the crystallinity of the coating and more defects in the coating. Smaller corrosion current density is related to the coating thickness. Compared with the potentiodynamic polarization curve of the coating in KF electrolyte, the corrosion potential of 0.129 mol/L KF coating was positive, and the corrosion current density was low, indicating that the corrosion resistance of the coating was the best. In the NH_4_F electrolyte, with the increase in NH_4_F concentration, the corrosion potential becomes negative, and the corrosion resistance of the coating decreases.

## 4. Discussion

**Figure 9:**
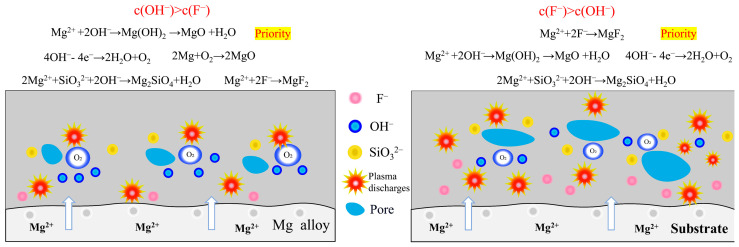
Coating Formation Mechanism of MAO Based on the Solubility Product Rule.

Figure 9 is a schematic diagram of the film formation mechanism of MAO in electrolytes with different fluorine additives. Table 6 shows the minimum concentration of Mg^2+^ for the formation of MgF_2_ and Mg(OH)_2_ in electrolytes with different fluorine additives based on the solubility product rule. When considered in conjunction with Figure 1 and Table 1, it becomes evident that the breakdown and operating voltages during MAO are significantly influenced by the varying pH and conductivity of the KF and NH_4_F electrolytes. KF continued to join, the conductivity of the electrolyte increases, the ion migration rate is accelerated, the partial pressure of the electrolyte decreases, and the partial pressure of the specimen surface increases, so that the MAO reaction is more intense, the reaction produces a larger number of bubbles too late to spill out of the coating and is wrapped in the coating, the amount of molten material deposited in the coating decreases, and the thickness of the coating does not substantially growth. The thickness of the coating was not greatly improved (shown in Figure 4). In the KF electrolyte, due to the high concentration of OH^−^, while the Ksp of MgF_2_ and Mg(OH)_2_ are 5.16 × 10^−11^ and 5.61 × 10^−12^, respectively, and OH^−^ preferred F^−^ reacts with Mg^2+^ in the KF electrolyte, and the coating-forming phases in the electrolyte are mainly MgO and Mg_2_SiO_4_ (shown in Figure 5 and Table 2). It can be said that the role of KF in the electrolyte is not fully exploited. According to the solubility product theory, we can know that in the sodium silicate base electrolyte, no matter how high the KF concentration is added, its film-forming phase is always dominated by magnesium oxide.

Increasing NH_4_F concentration in the electrolyte reduces conductivity and raises breakdown voltage during MAO processing. This hinders energy penetration through the formed passivation layer, generating excessive bubbles that promote molten material spillage. Consequently, coating thickness increases while internal densification decreases (Figure 4 and Figure 7), with molten material accumulating locally on the coating surface (Figure 6). At 0.129 mol/L NH_4_F concentration, partial F^−^ hydrolysis occurs while MgO and Mg_2_SiO_4_ remain dominant phases. When NH_4_F exceeds 0.129 mol/L with pH < 12.1, according to the solubility product theory, F^−^ preferentially reacts with Mg^2+^ over OH^−^, shifting the main coating phases from MgO and Mg_2_SiO_4_ to MgF_2_ (Figure 5 and Table 3).

## 5. Conclusions

1. In silicate-based electrolyte system, regardless of the variations in KF content, the main component of the MAO coatings formed on Mg alloy remained MgO along with a minor presence of magnesium silicate Mg_2_SiO_4_. This was because the introduction of KF not only elevated the F^−^ concentration in the electrolyte but also increased the OH^−^ concentration as a result of F^−^ hydrolysis. Based on the solubility product constants (Ksp) of MgO and MgF_2_, a relatively lower concentration of Mg^2+^ was sufficient for the formation of MgO. Hence, Mg^2+^ consistently exhibited preferential reactivity with OH^−^, leading to the formation of MgO. In the silicate-based electrolyte system, it was not feasible to prepare a MgF_2_ coating simply by increasing the KF concentration.
2. On the contrary, in NH_4_F electrolyte with a high pH, the OH^−^ preferred F− reacts with Mg^2+^, and the main coating-forming phases of the coating are MgO and Mg_2_SiO_4_. Conversely, in an electrolyte with increasing NH_4_F concentration, F^−^-preferred OH^−^ reacts with Mg^2+^, and the main coating-forming phases of the coating are transformed from MgO and Mg_2_SiO_4_ to MgF_2_. However, MgF_2_ coatings have many defects, with large surface pores, which actually lead to decreased corrosion resistance.

## Figures and Tables

**Table 1:** Basic properties of the electrolytes.

	KF		NH_4_F	
F^−^ (mol/L)	Conductivity (mS/cm)	pH	Conductivity (mS/cm)	pH
0.042	42.7	12.9	34.6	12.6
0.071	45.8	13.0	29.4	12.6
0.1	47.6	13.0	26.3	12.2
0.129	50.9	13.1	23.2	12.1
0.158	54.1	13.2	20.7	11.9
0.187	56.6	13.3	18.7	11.6
0.216	57.9	13.3	19.2	10.3

**Table 2:** Elemental content of coating in KF electrolyte (wt.%).

KF (mol/L)	Content of Elements (wt.%)					
	Mg	F	Si	O	Na	K
0.042	44.23	0.94	20.39	32.94	1.19	0.31
0.129	43.72	3.15	17.82	31.92	1.98	1.41
0.216	42.67	5.43	15.53	31.05	2.85	2.47

**Table 3:** Elemental content of coating in NH_4_F electrolyte (wt.%).

NH_4_F (mol/L)	Content of Elements (wt.%)					
	Mg	F	Si	O	Na	N
0.042	41.95	1.39	18.91	35.25	1.22	1.28
0.129	39.01	6.73	17.89	32.62	2.45	1.30
0.216	35.81	20.91	14.53	23.51	3.72	1.52

**Table 4:** Surface porosity and average pore size of coatings.

	KF		NH_4_F	
F^−^ (mol/L)	Average Size (μm)	Porosity	Average Size (μm)	Porosity
0.042	7.126	5.027	7.756	4.007
0.129	5.928	4.342	6.691	3.582
0.216	6.86	4.274	19.211	6.065

**Table 5:** Polarization curves parameters of the coatings.

Specimens	*E_corr_* (mV vs. Ag/AgCl)	*i_corr_* (A/cm^2^)
0.042 mol/L KF	−1341	3.93 × 10^−8^
0.129 mol/L KF	−1327	8.91 × 10^−9^
0.216 mol/L KF	−1386	6.61 × 10^−8^
0.042 mol/L NH_4_F	−1332	2.08 × 10^−8^
0.129 mol/L NH_4_F	−1425	2.65 × 10^−8^
0.216 mol/L NH_4_F	−1486	2.45 × 10^−9^

**Table 6:** Ionic coating formation concentration based on solubility product rule.

Specimens	OH^−^ (mol/L)	Ksp [Mg(OH)_2_] c (Mg^2+^) (mol/L)	Ksp [MgF_2_] c (Mg^2+^) (mol/L)
0.042 mol/L KF	6.31 × 10^−2^	8.89 × 10^−10^	2.92 × 10^−8^
0.129 mol/L KF	7.74 × 10^−2^	3.38 × 10^−10^	3.10 × 10^−9^
0.216 mol/L KF	2.09 × 10^−1^	1.29 × 10^−10^	1.11 × 10^−9^
0.042 mol/L NH_4_F	4.27 × 10^−2^	3.08 × 10^−9^	2.92 × 10^−8^
0.129 mol/L NH_4_F	1.26 × 10^−2^	3.54 × 10^−8^	3.10 × 10^−9^
0.216 mol/L NH_4_F	1.81 × 10^−4^	1.69 × 10^−4^	1.11 × 10^−9^

## Data Availability

The original contributions presented in this study are included in the article. Further inquiries can be directed to the corresponding author.
